# A new self-management engagement scale for haemodialysis patients (PRIESM CKD-HD): development and validation

**DOI:** 10.1093/ckj/sfaf364

**Published:** 2025-12-04

**Authors:** Helen Munro Wild, David Wellsted, Andrew Davenport, Paul Cockwell, Ken Farrington

**Affiliations:** Department of Psychology, Sport and Geography, School of Health, Medicine and Life Sciences, University of Hertfordshire, Hertfordshire, UK; Department of Psychology, Sport and Geography, School of Health, Medicine and Life Sciences, University of Hertfordshire, Hertfordshire, UK; UCL Department of Renal Medicine, University College London, London, UK; University Hospitals Birmingham NHS Foundation Trust, Department of Renal Medicine, Birmingham, UK; Department of Psychology, Sport and Geography, School of Health, Medicine and Life Sciences, University of Hertfordshire, Hertfordshire, UK; Renal Unit, Lister Hospital, Stevenage, UK

**Keywords:** chronic kidney disease patient reported outcome measure (CKD PROM), haemodialysis, psychosocial, scale development, self-management

## Abstract

**Background:**

Self-management should be better defined within kidney research and clinical practice. A focus on dialysis related tasks and adherence continues to be central to current measures. PRIESM CKD-HD (Patient Reported Instrument for Engagement in Self-Management for people with Chronic Kidney Disease, receiving Haemodialysis) aligns with a biopsychosocial model and measures engagement in self-management by people receiving haemodialysis (HD). This provides the opportunity to identify gaps and foster discussion about targeted support.

**Methods:**

Scale development was undertaken using a sequential mixed-methods approach, comprising literature review, qualitative interviews with HD patients (*n* = 27), expert consensus (*n* = 19) and cognitive testing (*n* = 11). A large-scale survey (*n* = 363) across three kidney centres was conducted to validate the scale, with subgroup follow-up 4–6 weeks later. Exploratory factor analyses were conducted to assess model fit, reliability and validity.

**Results:**

PRIESM CKD-HD is a valid and reliable measurement. Exploratory factor analysis indicated a three-factor model consisting of daily managing and impact (α = 0.91), communication (α = 0.84) and clinical care (α = 0.66). Model statistics, χ^2^ = 703, 296 df, *P* < .001, CFI = 0.87, TLI = 0.86, RMSEA = 0.07, SRMR = 0.07, overall α = 0.92. Correlation with measures of depression [r(328) = –0.74, *P* < .001] and self-efficacy [r(337) = 0.75, *P* < .001] provided further evidence of validity. Test–retest reliability r = 0.84, *P* < .001, indicates good linear agreement over time (*n* = 95).

**Conclusions:**

PRIESM CKD-HD is the first measure of self-management that reframes the concept from the patient perspective and incorporates the psychosocial aspects of treatment impact and daily managing for HD patients. Findings suggest the perception that not all individuals wish to self-manage should be refuted.

## GRAPHICAL ABSTRACT

**GRAPHICAL ABSTRACT:**
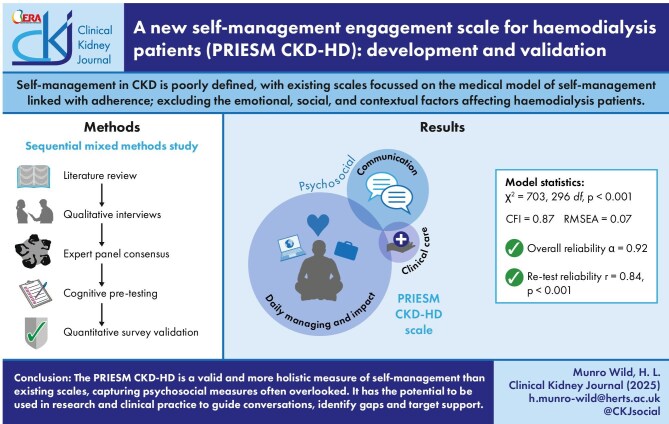

KEY LEARNING POINTS **What was known:**

- Self-management is a contested concept, and the kidney community has been slow to define and operationalise it in a way meaningful for people with kidney disease; current scales rely on the medical model of self-management linked with adherence and compliance.
- Through the introduction of a broader concept of self-management, a recognition of the diversity in the approaches, strategies and ways one might manage the impact of a life accompanied by illness may be better understood in the clinical setting.
- The development of PRIESM CKD-HD (Patient Reported Instrument for Engagement in Self-Management for people with Chronic Kidney Disease, receiving Haemodialysis) was designed to fill a gap in existing measures; most other kidney-specific measures are narrow in definition, lack relevance (not a haemodialysis sample) or generalisability (not validated in a UK population), and fail to meet the criteria that are a prerequisite for good scale development.

**This study adds:**

- We have developed a more holistic measure of self-management to better assess current engagement and support needs using a mixed method, consensus approach, involving a community of experts and people with lived experience; the measure is underpinned by a biopsychosocial model incorporating clinical (biological), social and emotional domains.
- The measure has strong psychometric properties; the model of best fit was found to be a three-factor, 26-item model with items grouped to subscales, daily managing and impact, communication, and clinical care; it correlates with two other similar constructs, depression and self-efficacy, and shows variation by patient characteristics.
- PRIESM CKD-HD includes items that relate to the behaviours necessary for the maintenance of emotional and social wellbeing and could be used in research and at an individual level in the clinical setting to guide more holistic conversations with patients and target support.
- Understanding how treatment and illness impact functioning and psychosocial wellbeing and considering the individual experience of managing and engaging in health is likely to improve the communication between patients and healthcare providers.

**Potential impact:**

- If implemented into clinical practice, the scale could provide a systematic way of discussing psychosocial wellbeing, normalise conversations around the patient context beyond the dialysis chair and improve collegiate working across multidisciplinary renal teams—potentially improving person-valued outcomes such as quality of life, as well as adherence and associated clinical outcomes.
- In research, PRIESM CKD-HD provides the potential for consistent, appropriate and effective evaluation of any self-management intervention and detects change over time.

## INTRODUCTION

End-stage kidney disease requires complex and time-consuming treatment, imposing significant burden, leading to poor mental health and having a significant impact on quality of life. Living with the disease requires strategies to meet and cope with the demands of daily living.

Research in self-management has increased steadily over two decades [in a PubMed search conducted 4 February 2024, using terms ‘psychosocial’ AND ‘kidney’, adults only, January 2022 to present day, 311 papers were identified; this is almost equivocal to the number published in the decade between 1995 and 2005 (*n* = 298)]. Self-efficacy, self-care [1], patient activation [2], shared care [3] and adherence [4] are often conflated with self-management or used as proxy measures (Supplementary data, Table S1). Ambiguity remains around definition and measurement in research, and the complex associations between for example, social support, depression, treatment adherence and involvement in decision-making. Whilst there is evidence linking many psychosocial elements to each other, and to self-managing [5–7], the underpinning mechanisms remain opaque [8–10]. The concept of self-management in research, is often only contextualized within the specific intervention being investigated.

Unlike other concepts, self-management is unique as it represents neither an internal state nor an intention, and goes beyond treatment-related tasks. Self-management is critically important beyond the clinic; it is a crucial part of daily care, particularly for those receiving haemodialysis (HD) [11]. Effective engagement in self-management goes beyond the physical and medical, extending into the social and emotional (termed psychosocial) [12].

This perspective counteracts over-medicalization of care and focuses on adaptation to illness, and impact on emotional wellbeing [13]. Self-management activities involving social and emotional strategies may facilitate better quality of life [14], improve capacity to engage in care [15] and have a significant impact on clinical outcomes [16].

Most existing scales rely on the biological model of self-management defined by adherence and clinical outcomes. No current measure adequately captures psychosocial elements, and few were developed with involvement by patients or validated in a UK HD population. We aimed to develop a measure of self-management [Patient Reported Instrument for Engagement in Self-Management for people with Chronic Kidney Disease, receiving Haemodialysis (PRIESM CKD-HD)], underpinned by patient experience, which aligns with a biopsychosocial model [17, 18].

## MATERIALS AND METHODS

**Figure 1:**
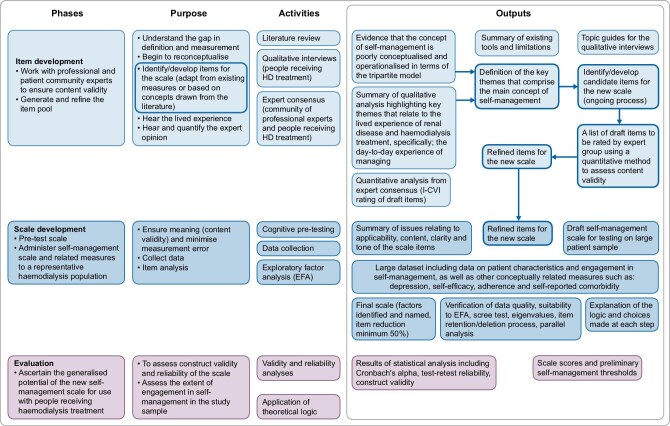
Self-management scale development framework.

The study was approved by East Midlands and Leicester South NHS Research Ethics Committee, REC reference: 17/EM/0451, and followed an exploratory sequential four-phase mixed methods design (Fig. 1) using best practice [19–21]. Reframing the concept of self-management in terms of what it means to patients underpinned the methodological approach. Participants had received HD, for ≥3 months (in-centre, satellite and home). All study phases were conducted across three sites: Lister Hospital, Stevenage, Royal Free Hospital, London, and University Hospital, Birmingham.

### Item development

Item development incorporated both inductive and deductive approaches, i.e. evidence from existing literature and qualitative data from interviews with the population of interest and item review by ‘experts’, i.e. professionals and people with lived experience in the kidney community, which aligns with this approach [22]. This design improves inclusivity and diversity of perspectives, which may strengthen validity of the scales.

A critical literature review was conducted to identify existing self-management measures and related psychosocial factors. PubMed, the Cochrane review and PsychNet were searched, between 1998 and 2018, using key terms including ‘self-management’ OR ‘self-care’ AND ‘ESRD’ OR ‘dialysis’. An over inclusive approach was adopted to ensure all important psychosocial dimensions were captured.

In-depth interviews with recipients of HD were undertaken to develop themes derived from lived experience relating to managing disease, treatment and life. The topic guide (Supplementary data, Fig. S1) was informed by the literature review. Purposive sampling ensured representation of the wider UK dialysis population [23], specifically, heterogeneity in age, sex, ethnicity and number of years on dialysis. Oversampling of South Asian and Black people ensured representation of those disproportionately burdened with CKD [24]. Interviews were conducted by H.M.W. at in-centre unit locations during dialysis, and at home for two home HD patients. Data were analysed by four researchers according to the Braun and Clark (2006) thematic analysis framework [25]. Each coded the same six scripts to reach a broad consensus on coding. This coding frame was then broadly applied to the remaining scripts by H.M.W.

Themes emerging from the empirical data were linked to evidence from the literature, to generate the scale items in conjunction with an expert panel and the wider research team. Candidate items were generated by H.M.W., and some were adapted from existing measures. The pool of candidate items was reviewed by the research team and then evaluated by an expert panel, drawn from existing networks with professional, clinical or research interests in kidney disease and self-management, and people receiving HD. Consensus was assessed in a survey using item content validity index (I-CVI) rating for measuring proportional agreement. Each expert rated items on a scale of 1 to 4 (1 not relevant, 4 highly relevant). I-CVI was the proportion of experts rating 3 or 4 [26]. Items were deemed relevant and considered for inclusion in the scale where the I-CVI was ≥0.78. Two rounds of I-CVI were required due to the volume of candidate items. The panel also assessed wording, simplicity, unambiguous meaning and tone.

### Scale development

Cognitive pre-testing helps assess whether the scale remains meaningful for the target user and that questions are easily understood, not ambiguous or difficult to answer [20]. The process used a combination of think-aloud and verbal probing. People receiving HD were asked to sense check scale items and response options. Interviews were conducted by H.M.W. Responses on formatting, item wording, response options and content were recorded and analysed.

The psychometric properties of the final draft version of the scale were tested as a 7-point Likert scale on a large, heterogenous cross-section of people receiving HD via a self-reported paper-and-pen survey, alongside other measures [a demographic and clinical characteristics questionnaire, the End-Stage Renal Disease Adherence Questionnaire (ESRD-AQ), PHQ-9 Depression Test Questionnaire and Self-Efficacy for Managing Chronic Disease Questionnaire (SEMCD-6)] included for the purpose of validation. Those consenting to follow-up were sent the scale plus a Change of Circumstances Questionnaire 4–6 weeks later, to assess instrument stability over time.

### Survey analyses

Item-total correlations, correlation between factors, strength and specificity of factor loading, communality and internal reliability (coefficient alpha) analyses were used to guide decision-making. Model fit was evaluated using standardized root mean square residual (SRMR), root mean square error of approximation (RMSEA), comparative fit index (CFI) and Tucker Lewis Index (TLI). Satisfactory fit was decided *a priori* as three of these four criteria being met, SRMR ≤0.10, and CFI and TLI ≥0.9 [22].

An initial cut-off criterion of 0.32 was used for factor loadings, increased to <0.5 for retention in the final model. Items with evidence of cross-loading between factors (<0.2 units difference), and those with adjusted item-total correlations <0.30, were also considered for omission. Internal consistency was assessed using Cronbach’s alpha for all subscales and the final version of the full scale. An alpha co-efficient of ≥0.80 was considered *a priori* as an acceptable threshold for reliability [27]. Instrument stability was assessed using test–retest reliability at 4–6 weeks. Intraclass correlation coefficient was applied to baseline and follow-up surveys.

Both scale average and domain scores were generated and used to test convergent validity using data from measures of adherence (ESRD-AQ) [28], depression (PHQ-9) [29] and self-efficacy (SEMCD-6) [30]. An inverse correlation between self-management score and depression, and a positive correlation with adherence and self-efficacy was deemed plausible based on theoretical frameworks and patterns observed in the literature.

Analyses were conducted using STATA IC 14 (StataCorp 2015, Stata Statistical Software: Release 14; StataCorp LP, College Station, TX, USA).

## RESULTS

The following results focus on the quantitative evaluation of the PRIESM CKD-HD. Results of the literature review are presented in more detail elsewhere [31], an overview is provided below.

### Item development

#### Literature review

**Figure 2:**
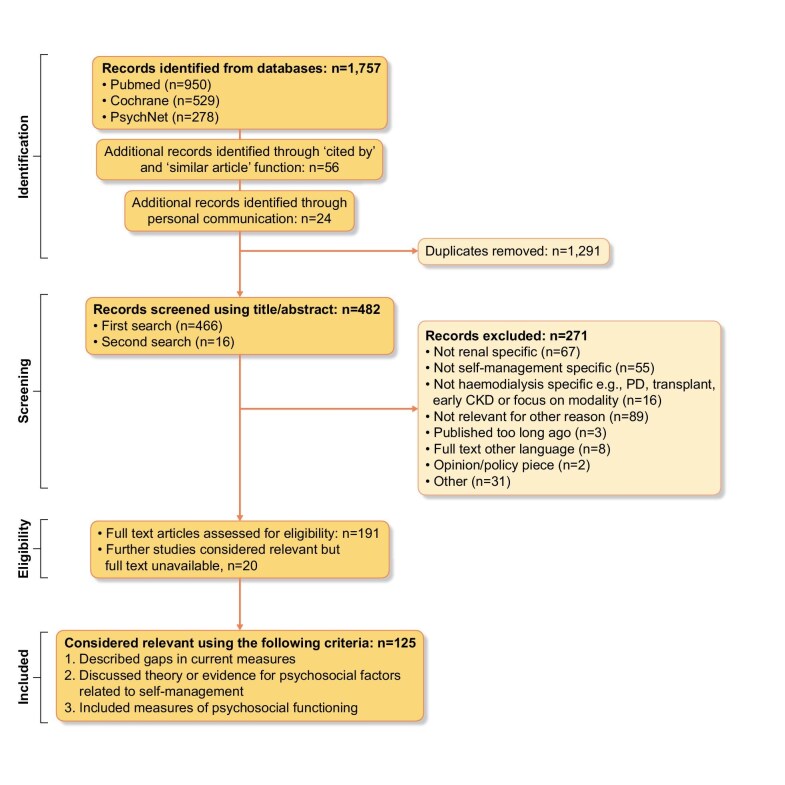
PRISMA flow diagram.

The literature review identified 482 articles that underwent screening (Fig. 2). The main search (April–June 2018) informed most of the scale development work. A total of 125 articles were deemed relevant and underwent data extraction. Key data were collated including aims and research question, study design, psychosocial and clinical outcomes, and self-management interventions.

Themes relating to or describing self-management identified in the literature informed the interview guide for the in-depth interviews. They included social support, perceptions of control, severity of symptoms, information/knowledge, communication, acceptance and coping strategies. Table 1 shows existing published self-management scales for kidney disease. Only four measures have been validated or used with samples including those receiving HD, plus a further two published since the development of PRIESM CKD-HD [32, 33], and none of the scales has been validated in a UK or European population which makes generalisability of the scale to such populations less certain. The inclusion of psychosocial factors such as those highlighted in the wider literature review is limited.

**Table 1:** Published self-management scales for kidney disease.

**Measure**	**Reference, country**	**Condition**	**Study population**	**Items/domains**	**Limitations**
Kidney Disease Behaviour Inventory (KDBI) was developed using the Summary of Diabetes Self-Care Activities (SDSCA) measure	Toobert *et al*. (2000), USA	Type 2 diabetes (SDSCA), then CKD patients, stage 3–5	Seven studies in type 2 diabetes populations (*n* ∼2000 total). Adapted for kidney patients and used with both HD (*n* = 146) and non-HD CKD (*n* = 237)	KDBI 16-item scale [described by Wild *et al*. (2017)]	High correlation with PKDSMS. Difficult to evaluate as no specific paper found about development of KDBI
				• Specific self-care activities	
				• Adherence relating to diet, medication, attending appointments and dialysis treatment	
Behaviours on Haemodialysis Scale (BHDS)	Curtin *et al*. (2004), USA	End-stage renal disease	In-centre HD patients (*n* = 372, 17 centres)	37-item scale across 8 domains	Wording, questions with multiple clauses. Recall period 6 months. Only applicable to in-centre HD. Limited psychosocial elements
				• Suggestions to providers	
				• Self-care during HD	
				• Information seeking	
				• Use of alternative therapies	
				• Selective symptom management	
				• Assertive self-advocacy	
				• Impression management	
				• Shared responsibility in care	
HD Self-Management Instrument (HD-SMI)	Song and Lin (2009), Taiwan, also cited by Cha and Kang (2017), Korea	End-stage renal disease	Developed and tested using a HD population attending 4 hospital centres (*n* = 196). Later translated and validated as a Korean version also on HD patients (*n* = 215)	Unclear how many items in the final scale	Developed using a Taiwanese HD population and translated into Korean in a second study. Full texts unavailable in English
				• Problem solving and communication	
				• Fluid and weight control	
				• Diet and HD	
				• Self-advocacy and emotion control	
Partners In Health (PIH)^®^ instrument	Walker *et al*. (2013), New Zealand	CKD	Recruited from two primary care practices serving areas of high socioeconomic deprivation included if at ‘high risk of CKD progression’ (*n* = 52)	13-item scale	Developed as more generic tool to look at treatment burden in patients with multimorbidity. Results of main study not published
				• Knowledge of health condition/medications/treatment	
				• Medication compliance	
				• Adherence to a healthy lifestyle	
CKD self-management instrument (CKD-SM)	Lin *et al*. (2012), Southern Taiwan	Early-stage CKD	Mandarin or Taiwanese speaking adults with CKD recruited from two medical centres and one regional hospital in Southern Taiwan (*n* = 252)	29-item scale	∼90% of participants were stage 2 or 3 CKD. Validated in a Taiwanese renal population
				• Self-integration	
				• Problem solving	
				• Seeking social support	
				• Adherence	
CKD Self-Management scale (CKD SM)	Johnson *et al*. (2016), USA	CKD stage 3	Sample recruited from 5 primary care, 3 nephrology clinics and 1 dialysis centre in two Midwestern cities in USA (*n* = 85)	15-item scale; no information about specific items or domains	Tested in patients CKD stage 3. Original scale unpublished. Paper does not include scale items. Just mentions 1 item ‘I understand my fluid restriction’
Perceived Kidney/Dialysis Self-Management Scale (PKDSMS)	Wild *et al*. (2017), USA	CKD stages 3–5	Kidney patients on HD (*n* = 146) and non-HD CKD (*n* = 237). Collected as separate samples	8-item scale• Illness perceptions• Self-identity	Measure of self-efficacy or perceived competency (internal states) rather than self-management behaviours
CKD Self-Care scale (CKDSC)	Wang *et al*. (2019), Taiwan. Originally cited Wang *et al*. (2016) but full text unavailable	CKD (stages 1–5)	Convenience sample of CKD patients, excluded those on renal replacement therapy or who had received a transplant (*n* = 449)	16-item scale	Only validated in a Taiwanese population. Assesses patient perceived self-care
				• Medication adherence	
				• Diet control	
				• Exercise	
				• Smoking behaviour	
				• Blood pressure	
**Measures published since the development of the PRIESM CKD-HD**					
Strategies Used by People to Promote Health (SUPPH)	Ibelo *et al*. (2022), Canada, Lev and Owen (1996), USA	HD population. Originally developed with cancer patients receiving chemotherapy	Outpatients receiving maintenance on HD in 7 centres in Calgary and Alberta (*n* = 50). Adult cancer patient receiving chemotherapy for cancer in ambulatory settings (*n* = 114)	Three dimensions, 29-item self-report scale, based on self-efficacy theory, that measures self-care self-efficacy. (i) Positive attitude; (ii) stress reduction; (iii) making decisions	A measure of ‘self-care self-efficacy’ rather than self-management. Despite its generality and focus on confidence, it does contain some important psychosocial elements, treatment decisions, meaningful life, anxiety and stress
CAPABLE	Devia *et al*. (2022), Colombia	Incident peritoneal patients	Described as peritoneal candidates (*n* = 20) attending the Baxter Renal Care Services, Colombia. 18+ years in the early phase of peritoneal dialysis training	The instrument has 3 domains and 16 items. The three domains are sensory, cognitive and motor capacity	Small sample. The scale has not been developed as quantitative scale but an inventory. Its focus is on self-care tasks related to treatment and the scale does not contain any psychosocial elements
Chronic Illness Self-Management (CISM)	Ngai *et al*. (2020), Hong Kong, Uğuz and Doğan (2023), Turkey	Chronic illness and then a HD population	Territory-wide study recruiting 12–45 years with chronic illness (*n* = 497). Patients treated at 3 HD centres in Izmir (*n* = 261)	The 5-point Likert scale consists of 21 items and 4 sub dimensions: self-stigma, coping with stigma, healthcare efficiency and treatment implementation	Initially developed as a generalisable scale aimed at a younger population with chronic disease. Validated in a specific sample, Chinese context of Hong Kong society, then in a Turkish sample of HD patients

#### Qualitative interviews

Twenty-seven people receiving HD treatment were interviewed (Table 2). Emerging themes were grouped into four elements (Table 3, illustrative quotes provided in Supplementary data, Table S2): experience, behaviours, perceptions and others (family, peers, healthcare providers). Most themes were unrelated to clinical care during dialysis. The most frequent were coping strategies, daily managing and communication with, and support from, the renal team.

**Table 2:** Participant characteristics (qualitative interviews).

**Characteristics**	**Respondents (*n* = 27)**	**Lister (*n* = 11)**	**RFH (*n* = 6)**	**UHB (*n* = 10)**
Mean age (range), years	58 (24–89)	60 (35–86)	62 (40–74)	54 (24–89)
Age groups, *n* (%)				
20–39 years	5 (18.5)	2 (16.7)	0 (0)	3 (30.0)
40–59 years	9 (33.3)	2 (16.7)	3 (50.0)	4 (40.0)
60–74 years	8 (29.6)	4 (36.4)	3 (50.0)	1 (10.0)
≥75 years	5 (18.5)	3 (27.3)	0 (0)	2 (20.0)
Sex, *n* (%)				
Male	18 (66.7)	8 (72.7)	4 (66.7)	6 (60.0)
Female	9 (33.3)	3 (27.3)	2 (33.3)	4 (4)
Ethnicity (broad), *n* (%)				
White British	12 (44.4)	2 (18.2)	3 (50.0)	7 (70.0)
Asian/Asian British	4 (14.8)	2 (18.2)	1 (16.7)	1 (10.0)
Black/African/Caribbean/British	9 (33.3)	6 (54.5)	2 (33.3)	1 (10.0)
Mixed/multiple ethnic groups	1 (3.7)	0 (0)	0 (0)	1 (10.0)
Missing	1 (3.7)	1 (9.1)	0 (0)	0 (0)
Location, *n* (%)				
In-hospital	4 (14.8)	4 (36.4)	0 (0)	0 (0)
Satellite	21 (77.8)	6 (54.5)	6 (100)	9 (90.0)
Home	2 (7.4)	1 (9.1)	0 (0)	1 (10.0)
Time on dialysis, *n* (%)				
<12 months	10 (37.0)	5 (45.5)	2 (33.3)	3 (30.0)
12–24 months	4 (14.8)	2 (18.2)	0 (0)	2 (20.0)
25–36 months	9 (33.3)	2 (18.2)	3 (50.0)	4 (40.0)
>3 year	4 (14.8)	2 (18.2)	1 (16.7)	1 (10.0)

**Table 3:** Key themes from the qualitative analysis.

**Element**	**Themes**	**Description**
Experience	Dialysis process	The demands of dialysis such as the restrictions around 3–4 days a week schedules, the time lost, complications and the barriers to taking holidays
	Symptoms	The experience of both physical and/or emotional symptoms, the burden and severity and how these impacts on self-managing behaviours. Includes the strategies that are used to cope with or prevent symptoms
	Other health conditions	The extent to which multimorbidity complicates managing of kidney disease, particularly when multiple care providers are involved, and treatment recommendations/appointments may conflict. How does it impact on day-to-day priority setting and the capacity to engage with tasks of daily living?
	Kidney transplant	Perceptions of the barriers to being on the transplant list, the experience of waiting, understanding how decisions are made and expectations
Behaviours	Management of daily tasks	Managing psychological responses to illness as well as practical aspects of managing illness either independently or with support from others, within the context of relationships, goals, values and life as usual
	Knowledge	If, when and how patients seek information and how they use it. In what context is information sought and for what purpose, may be reassurance or in decision-making about treatment options, etc.
	Coping strategies	Emotion and task focussed engagement strategies that are used to manage the illness within the context of everyday life
	Meaningful life	Behaviours that help achieve a sense of balance. Maintaining activities that impact on wellbeing and provide a sense of purpose. Examples of how illness has been integrated into daily living a way that enables a person to maintain purpose and meaning
Perceptions	Beliefs about risks and consequences	Perceptions about the health risks linked to certain behaviours and adherence. Understanding the impact of behaviour in the long and short term
	Illness perceptions	Making sense of illness, its origins, and consequences. Impact of illness perceptions on physical function, adaptation and acceptance, and the impact of social situations
	Self-identity	The intersection with illness and self and the degree to which illness is integrated into identity. Relational roles and social identity form core aspects of identity
	Empowerment and control	Approaches to change, coping and enablers of self-management. A sense of control over the illness that is sufficient to allow involvement in the management of it if wanted. An ability to let go or accept things that cannot be controlled
	The future	Maintaining hope and anticipation for good things still to come. Anticipation and preparedness for the future, and reflections on a life lived
Others	Impact on family	The degree to which family members provide both task-based and emotional support. The impact of this on those caring and the person supported. Examples, enabling self-management, increased family conflict, feelings of being a burden, or where support is perceived as negative and may affect the patient’s ability to self-manage
	Healthcare providers	Support offered by clinical staff to promote and support self-management that goes beyond information sharing and medical support of dialysis to wider goal setting, problem-solving support, joint decision-making and more holistic support. Patient’s perception of the quality, availability and experience of support

Theory and evidence from the literature review was linked with the empirical evidence generated by the qualitative interviews to underpin item development. The themes from the qualitative work were used alongside the literature to generate items for the scale and reiterate the themes.

#### Finding consensus

Instructions and the pool of candidate items within 17 themes were circulated to the members via email; Round 1 included *n* = 155 items, and Round 2 *n* = 89 items. All but one of the 18 members of the expert panel completed both rounds of I-CVI rating. In Round 1, 72 out of 155 (45.5%) items met or exceeded the ≥0.78 threshold and were retained. Eight items had an I-CVI = 1.0, indicating high consensus regarding relevance. The themes in which the greatest proportion of items were ≥0.78 was ‘managing day to day’ (10/14) (Table 4).

**Table 4:** I-CVI process.

	**I-CVI, Round 1**		**I-CVI, Round 2**		
**Theme**	**Included**	**Retained**	**Included**	**Retained**	**60-item scale**
Acceptance	10	6	4	4	4
Communication	9	4	5	4	3
Control	8	2	4	3	3
Coping strategies	16	6	8	6	5
General health/multimorbidity	7	2	6	4	5
Hope	8	3	4	2	2
Illness perceptions and identity	12	5	5	3	3
Impact on family	11	6	5	5	5
Information/knowledge	7	4	6	5	3
Managing day to day	14	10	8	5	3
Meaningful life	8	3	4	4	3
Selfcare	0	-	5	5	6
Social support	8	2	3	3	2
Support (MDT)	8	5	7	4	3
Symptoms	9	5	6	6	4
Treatment decisions	13	4	5	5	4
Work	7	5	4	3	2
Total number of items	155	72	89	71	60

MDT, multidisciplinary team.

In Round 2, 18 items were dropped (leaving 71 items). Table 4 illustrates how the themes translated into the scale and continued to be represented throughout development. This process also led to changes to wording and response options of items (see Supplementary data, Table S3 for an example). A further 5 items were then removed by the core research team due to duplication (leaving 66 items).

#### Cognitive pre-testing

A draft version of the scale was pre-tested with 11 individuals (mean age 58 years, range 24–81 years, 7 males, 3 Asian/Asian British). Most items were found to be relevant to their personal circumstances. All respondents were broadly happy with the tone of the questions. Recommendations for item amendment based on the cognitive interviews were reviewed and discussed with the research group following which 6 items were removed (Supplementary data, Table S4, giving a scale with 60 items). Forty items were reworded between the second round of I-CVI and the administration of the survey.

#### Survey administration

Over 2000 packs, which included the 60-item self-management scale [31] and other measures described in the methods, were sent out to support recruitment. Data were analysed for 363 patients across the three kidney centres. Age, sex and ethnicity of respondents were broadly representative of the UK renal registry HD population [23]. Fifty-two percent were married or living with a partner. Seventy-six percent reported one or more other long-term conditions—diabetes (36.6%), arthritis (28.9%), heart disease (28.1%), depression (15.7%) and cancer (13%), with 39.4% having two or more. Some 21% required some help completing the scale. Participant characteristics of the retest subsample (*n* = 95) were similar (Table 5).

**Table 5:** Demographic and clinical characteristics in baseline and test–retest surveys.

**Characteristic** **^a^**	**Baseline survey sample** **^b^**	**Test retest sample**
Kidney centre		
University Hospital Birmingham	138 (38.0)	61 (64.2)
Lister Hospital	138 (38.0)	13 (13.7)
Royal Free Hospital	87 (24.0)	21 (22.1)
Age, years		
20–39	27 (7.5)	6 (6.3)
40–59	115 (31.8)	26 (27.4)
60–74	136 (37.6)	36 (37.9)
75 or older	84 (23.2)	27 (28.4)
Sex		
Men	233 (64.4)	56 (58.9)
Women	129 (35.6)	49 (41.1)
Ethnicity		
White	250 (69.3)	73 (76.8)
Asian and Asian British	42 (11.6)	11 (11.6)
Black African Caribbean and Black British	49 (13.6)	67 (6.3)
Mixed and multiple ethnic groups	12 (3.6)	2 (2.1)
Other ethnic group	7 (1.94)	3 (3.2)
Time since diagnosis, years		
Within last year	19 (5.3)	7 (7.4)
1–5	118 (33.2)	25 (26.3)
6–10	70 (19.7)	17 (17.9)
≥10	128 (36.0)	41 (43.2)
Since birth or childhood	21 (5.9)	4 (1.1)
Length of time on dialysis, years		
<1	65 (18.0)	18 (19.0)
1–2 (inclusive)	97 (26.8)	18 (19.0)
2–3	42 (11.6)	10 (10.5)
3–4	40 (11.1)	9 (9.5)
4–5	30 (8.3)	8 (8.4)
5–10	58 (16.0)	19 (20.0)
≥10	30 (8.3)	13 (13.7)

a Data are presented as *n* (%).

b Data were missing for 13/17 demographic and clinical characteristics, range missing 0.3%–9%. For 15/17, missing data <3%. Proportion missing data was highest for marital status and education.

#### Item-level descriptive statistics

Overall, missing data across all questionnaires was low; most items in the PRIESM CKD-HD had missing data ≤5% (58/60). The greatest proportion missing was 6.1% for items 18 and 39 (‘I adjust my phosphate binder to the size of my meal’ and ‘I am confident in the advice my GP gives me about my kidney disease’). Selection of the ‘don’t know’ response ranged from 0% to 4.57% and for ‘not applicable’, 0%–17.9%. Mean scores for each item ranged from 2.56 to 6.58 [standard deviation (SD) = 0.99–2.48]. Item total correlations were between 0.18 and 0.19. Participants reporting the lowest or highest item score (floor, or ceiling effects) ranged from 1.1% to 40.4% and 10.9% to 76.6%, respectively. There was some evidence of a ceiling effect; ≥50% of participants reported the highest score for 13 of 60 items. For full descriptive statistics see Supplementary data, Table S5.

#### Exploratory factor analysis

**Figure 3:**
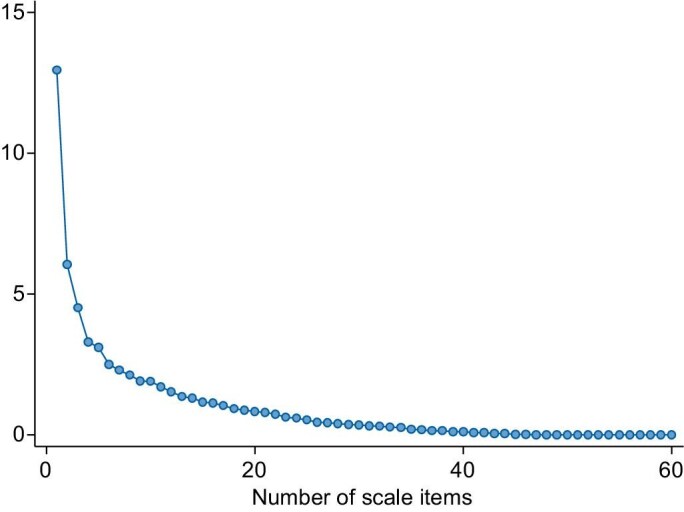
Scree plot of eigenvalues after factor (all 60 items).

Exploratory factor analysis using varimax orthogonal rotation was applied without a forced structure to the sample data (Supplementary data, Table S6, *n* = 363). The Kaiser-Meyer-Olkin value indicated adequate sampling (0.87), and Bartlett test of sphericity was significant (χ^2^ = 7253.886, df = 1770, *P* < .001), indicating correlation of variables. Cronbach’s alpha coefficient for internal consistency reliability was 0.92, indicating very good reliability. In conjunction with the eigenvalues, the scree plot was used to determine how many factors best fit the data. The steep drop and obvious break at factor 4 (Fig. 3) led to an initial four-factor model. The structure and strength of factor loadings were also considered, and parameterized as 3, rather than 4, due to high correlation between factors 3 and 4 (>0.7). Twenty-two items of the 60 were dropped.

Model iterations (Supplementary data, Tables S7 and S8) addressed several issues including cross-loading between factors (defined as <0.200 units difference between factors) and high inter-item correlation within factors (>0.50). The steps of the exploratory analysis showed that the fit indices remain relatively stable over the iterations but improved as items were removed. The output suggested a three-factor model approaching a reasonable fit (given the exploratory nature of the scale development and novel conceptualization of self-management), with all goodness of fit statistics close to the stated threshold (RMSEA and SRMR ≤0.10, and CFI and TLI ≥0.90); χ^2^ was 703 on 296 df, with associated RMSEA of 0.07, CFI = 0.87, TLI = 0.86 and SRMR = 0.07.

#### PRIESM CKD-HD: final model

The final PRIESM CKD-HD model comprised 26-items across three domains of ‘day to day managing and impact’ (14 items), ‘communication’ (8 items) and ‘clinical care’ (4 items). The scale with response options is shown in Supplementary data, Fig. S2. The model accounted for 93% of the variance in the sample data, with the 14 items in factor one explaining about 65% of the variance. Item total correlations vary from 0.30 to 0.32, indicating that the items correlate sufficiently without being repetitive in terms of measuring the broader self-management concept. Adequate unidimensionality was indicated by inter-item correlation <0.6. Eigenvalues for the forced three factor structure were 7.73, 2.29 and 1.05, respectively.

Factor loadings ranged from 0.49 to 0.75, 0.27 to 0.77, and 0.45 to 0.58 respectively. Items 8, 33 and 52 were retained in the scale despite issues as they were considered theoretically and were rated highly relevant in the consensus process. Oblique rotation using the oblimin method was run as a secondary check but made no difference to the structure of the final scale; model statistics, χ^2^ = 703, 296 df, *P* < .001, CFI = 0.87, TLI = 0.86, RMSEA = 0.07, SRMR = 0.07, overall α = 0.92.

#### Reliability

The overall scale and subscales showed reliable internal consistency (α = 0.91 overall, managing subscale α = 0.91, communication subscale α = 0.84 and clinical care subscale α = 0.66). Instrument stability was assessed using test–retest reliability at ≥4 weeks, and a Pearson correlation coefficient r(95) = 0.84 (*P* < .001) suggests good to linear agreement over time (at least in the short term).

#### Discriminant/convergent validity

**Figure 4:**
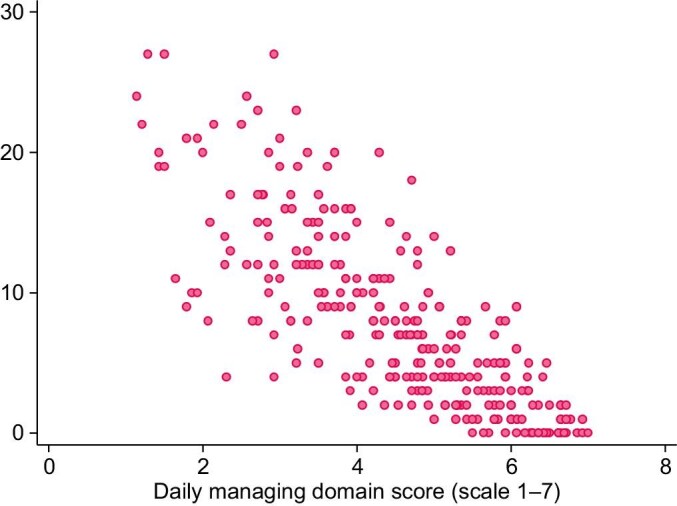
Managing domain of PRIESM CKD-HD and correlation with depression.

**Figure 5:**
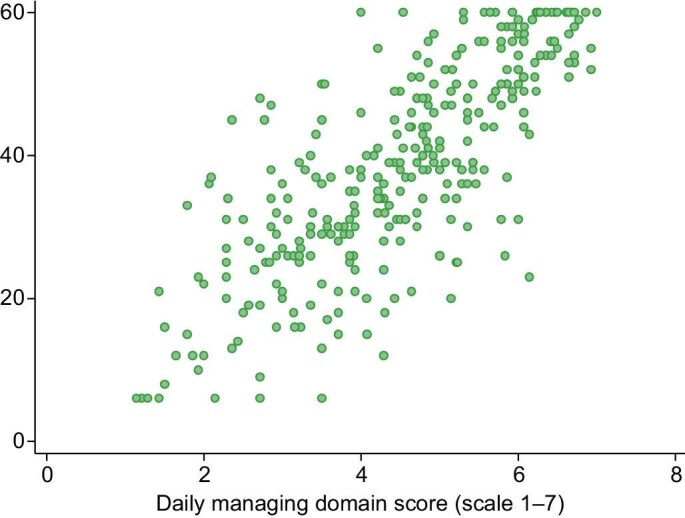
Managing domain of PRIESM CKD-HD and correlation with self-efficacy.

An inverse correlation was found between self-management [raw item scores from the scale were summed and the total score was standardized to a 0–7 range, with higher scores indicating greater engagement with self-management; composite scores for each of the three domains were generated in the same way; the overall self-management score mean was 5.02 (SD ± 1.08), the daily managing mean was 4.51 (SD = 1.36), for communication 5.83 (SD ± 1.15) and for clinical care 5.36 (SD ± 1.25)] and depression r(328) = –0.74 (*P* < .001) (Fig. 4), which increased to –0.80 (*P* < .001) using the daily managing and impact domain. There was a strong, positive correlation with self-efficacy r(337) = 0.75, *P* < .001 (Fig. 5) which increased slightly to 0.77 with the daily managing and impact domain. Correlation was poor between the adherence measure ESRD-AQ and PRIESM CKD-HD r(359) = 0.16 (*P* < .01). In shifting away from a more clinical definition of self-management it is perhaps unsurprising that adherence showed poor correlation with the scale. The relationship between the various measures of adherence and self-management is complex. As a proxy measure of adherence, there was some evidence of an association between better phosphate control and higher self-management score (see Supplementary data, Table S9).

## DISCUSSION

PRIESM CKD-HD was developed to broaden the conceptualization of self-management to align with the tripartite model of health. Development was underpinned by lived experience. The instrument allows patient engagement in self-management to be measured, to inform conversations about potential gaps in support. It was developed and validated in an HD population according to best practice principles. The best fitting model was a three-factor, 26-item model with three subscales: daily managing/impact, communication and clinical care. The subscales had acceptable Cronbach’s alpha scores and eigenvalues, and acceptable loadings on 23/26 items. Content, face validity and reliability were good and met accepted standards. PRIESM CKD-HD scores also correlated with those of depression and self-efficacy, supporting the relationship between these concepts and self-management [34–38]. The survey sample showed sufficient heterogeneity to make the scale generalizable to other HD populations.

As measured by the scale, daily managing/impact and communication account for more of the variance than clinical care, indicating that their relative importance to patients. Clinical care happens in a well-defined space and time, whereas the work of living and managing day-to-day is unremitting. Critically though, social and emotional factors have an impact on clinical care; not least around decision-making, modality choice, adherence, and experience of care and dialysis [39–42] as well as being intrinsically linked to quality of life [14]. These two main factors reflect a person-centred approach to scale development together with a redressing of the balance between medical, social and emotional needs, whilst remaining relevant in the clinical setting. Although the mechanisms driving some of the associations between elements such as social support, communication with healthcare providers, depression, adherence and treatment choices are not well understood, people receiving HD indicate psychosocial factors are of great importance.

There are some limitations. The self-management concept is complex, so despite good apparent psychometric properties, it is still possible to create incoherent constructs [43]. Whilst the model fit statistics are acceptable, less stringent thresholds were used in acknowledgement that the concept of self-management is both complex and contested within the wider research and clinical context. However, particularly in exploratory research, cut-off values are there to guide rather than applied rigidly. Three retained items (Item 8 ‘I feel more isolated than I did before I started dialysis’; Item 33 ‘I wasn’t as involved in the decision to start dialysis as I would have liked’; Item 52 ‘the renal team encourage me to be involved in my own care’) showed low factor loading and issues with cross-loading and uniqueness. However, each was rated highly relevant during the expert consensus process. Involvement in decision-making and feeling supported by the clinical care team are key components in self-management, this is supported by empirical evidence from the qualitative interviews and from the literature [44, 45]. Social isolation seems particularly pertinent since the COVID-19 pandemic, especially in chronic disease settings [46].

Strengths include use of qualitative patient data to understand the concept, and pre-testing of the scale, which reduce the potential for measurement error in the final scale. Use of multiple approaches optimizes data capture to inform item development and helps ensure content validity. Participants involved in both the qualitative and quantitative aspects of the study were broadly representative of the HD population in terms of age, sex and ethnicity.

The current structure of the scale is hypothesized using a single, albeit large, sample. Further studies would allow confirmatory factor analysis to explore whether the structure of the scale requires refinement perhaps in conjunction with other tools such as the Patient Activation Measure (PAM), used recently to measure the impact self-management interventions in kidney [47] as well as other long-term condition populations. Lightfoot *et al*. (2021) endorse the use of PAM for assessing patient activation in kidney patients, and similarly view activation as a precedent to self-management [48].

In conclusion, PRIESM CKD-HD could be used in clinical care and research to identify gaps in support and help patients engage with their illness and treatment to the extent that they wish. Interest in patient engagement is increasing and the importance of shared decision-making, and provider–patient communication are well-established though the concept of self-management still lacks consensus [49]. The impact of social factors on health continues to be underestimated [50] and is not prioritized in healthcare despite evidence that they are protective against mortality and can mediate behaviours linked to diet, fluid and medication adherence, as well as being intrinsically important to patients. Supported self-management requires identification of the broader context of the individual, what matters to them, where they are struggling and where there are gaps in support.

## Supplementary Material

sfaf364_Supplemental_File

## Data Availability

The authors confirm that the data supporting the findings of this study are available within the article and its supplementary materials and are available via the University of Hertfordshire Repository https://uhra.herts.ac.uk/id/eprint/25861/.

## References

[1] Richard AA, Shea K. Delineation of self-care and associated concepts. J Nurs Scholarsh 2011;43:255–64. 21884371 10.1111/j.1547-5069.2011.01404.x

[2] Hibbard JH, Mahoney ER, Stock R et al. Do increases in patient activation result in improved self-management behaviors? Health Serv Res 2007;42:1443–63. 10.1111/j.1475-6773.2006.00669.x 17610432 PMC1955271

[3] Moser A, Van Der Bruggen H, Widdershoven G et al. Self-management of type 2 diabetes mellitus: a qualitative investigation from the perspective of participants in a nurse-led, shared-care programme in the Netherlands. BMC Public Health 2008;8:91. 10.1186/1471-2458-8-91 18366665 PMC2292711

[4] Evangelista LS, Shinnick MA. What do we know about adherence and self-care? J Cardiovasc Nurs 2008;23:250–7. 10.1097/01.JCN.0000317428.98844.4d 18437067 PMC2880251

[5] Chen YC, Chang LC, Liu CY et al. The roles of social support and health literacy in self-management among patients with chronic kidney disease. J Nurs Scholarsh 2018;50:265–75. 10.1111/jnu.12377 29569423

[6] Griva K, Lam KFY, Nandakumar M et al. The effect of brief self-management intervention for hemodialysis patients (HED-SMART) on trajectories of depressive and anxious symptoms. J Psychosom Res 2018;113:37–44. 10.1016/j.jpsychores.2018.07.012 30190046

[7] Havas K, Douglas C, Bonner A. Person-centred care in chronic kidney disease: a cross-sectional study of patients’ desires for self-management support. BMC Nephrol 2017;18:1–9. 10.1186/s12882-016-0416-2 28086812 PMC5237219

[8] Cuevas H, Heitkemper E, Huang YC et al. A systematic review and meta-analysis of patient activation in people living with chronic conditions. Patient Educ Couns 2021;104:2200–12. 33610334 10.1016/j.pec.2021.02.016

[9] Leventhal H, Phillips LA, Burns ET. The Common-Sense Model of Self-regulation (CSM): a dynamic framework for understanding illness self-management. J Behav Med 2016;39:935–46. 10.1007/s10865-016-9782-2 27515801

[10] Jonkman NH, Schuurmans MJ, Jaarsma T et al. Self-management interventions: proposal and validation of a new operational definition. J Clin Epidemiol 2016;80:34–42. 27531245 10.1016/j.jclinepi.2016.08.001

[11] Miller WR, Lasiter S, Bartlett Ellis R. et al Chronic disease self-management: a hybrid conceptanalysis. Nurs Outlook 2015;63:154–61. 10.1016/j.outlook.2014.07.005 25241136 PMC4326608

[12] Corbin J, Strauss A. Managing chronic illness at home: three lines of work. Qual Sociol 1985;8:224–47.

[13] Ahmad N, Ellins J, Krelle H et al. Person-centred care: From ideas to action: Bringing together the evidence on shared decision making and self-management support. London, UK: The Health Foundation, 2014.

[14] Ibrahim N, Teo SSL, Din NC et al. The role of personality and social support in health-related quality of life in chronic kidney disease patients. PLoS One 2015;10:1–11. 10.1371/journal.pone.0129015 PMC4488553 26131714

[15] Narva AS, Norton JM, Boulware LE. Educating patients about CKD: the path to self-management and patient-centered care. Clin J Am Soc Nephrol 2016;11:694–703. 10.2215/CJN.07680715 26536899 PMC4822666

[16] Grady PA, Gough LL. Self-management: a comprehensive approach to management of chronic conditions. Public Health 2014;104:25–31. 10.2105/AJPH.2014.302041 PMC4103232 24922170

[17] Lorig KR, Ritter P, Stewart AL et al. Chronic disease self-management program 2-year health status and health care utilization outcomes. Med Care 2001;39:1217–23. http://journals.lww.com/lww-medicalcare 11606875 10.1097/00005650-200111000-00008

[18] Card AJ. The biopsychosociotechnical model: a systems-based framework for human-centered health improvement. Health Syst (Basingstoke) 2022;12:387–407. 38235298 10.1080/20476965.2022.2029584 PMC10791103

[19] Morgado FFR, Meireles JFF, Neves CM et al. Scale development: ten main limitations and recommendations to improve future research practices. Psicol Reflex Crit 2017;30:1–20. 32025957 10.1186/s41155-016-0057-1 PMC6966966

[20] Younas A, Porr C. A step-by-step approach to developing scales for survey research. Nurse Res 2018;26:14–19. 10.7748/nr.2018.e1585 30456936

[21] Boateng GO, Neilands TB, Frongillo EA et al. Best practices for developing and validating scales for health, social, and behavioral research: a primer. Front Public Health 2018;6:149. 29942800 10.3389/fpubh.2018.00149 PMC6004510

[22] Clark LA, Watson D. Constructing validity: new developments in creating objective measuring instruments. Psychol Assess 2019;31:1412–27. 10.1037/pas0000626 30896212 PMC6754793

[23] UK Renal Registry . UK Renal Registry 25th Annual Report—Data to 31/12/2021. Bristol, UK: Renal Registry/UK Kidney Association; 2023. Available from: https://ukkidney.org/audit-research/annual-report/25th-annual-report-data-31122021 (Accessed 29 September 2025).

[24] Mathur R, Dreyer G, Yaqoob MM et al. Ethnic differences in the progression of chronic kidney disease and risk of death in a UK diabetic population: an observational cohort study. BMJ Open 2018;8:e020145. 10.1136/bmjopen-2017-020145 PMC5875688 29593020

[25] Braun V, Clarke V. Using thematic analysis in psychology. Qual Res Psychol 2006;3:77–101. 10.1191/1478088706qp063oa

[26] Polit DF, Beck CT. The content validity index: are you sure you know what’s being reported? Critique and recommendations. Res Nurs Health 2006;29:489–97. 10.1002/nur.20147 16977646

[27] De Vellis RF. Scale Development: Theory and Applications, 2nd edn, Vol. 2. Thousand Oaks, CA: Sage Publications, 2003.

[28] Kim Y, Evangelista LS. Relationship between illness perceptions, treatment adherence, and clinical outcomes in patients on maintenance hemodialysis. Nephrol Nurs J 2010;37:271–80; quiz 281. 20629465 PMC3172671

[29] Kroenke K, Spitzer RL, Williams JBW. The PHQ-9: validity of a brief depression severity measure. J Gen Intern Med 2001;16:606–13. 11556941 10.1046/j.1525-1497.2001.016009606.x PMC1495268

[30] Lorig KR, Sobel DS, Ritter PL et al. Effect of a self-management program on patients with chronic disease. Eff Clin Pract 2001;4:256–62. 11769298

[31] Munro Wild HL. Development of a Unique Person-centred Self-Management Behaviour Scale for People on Haemodialysis. Hatfield: University of Hertfordshire, 2024.

[32] Ngai SSy, Cheung Ck, Ng Yh et al. Development and validation of the chronic illness self-management (CISM) scale: data from a young patient sample in Hong Kong. Child Youth Serv Rev 2020;114:105077. 10.1016/j.childyouth.2020.105077

[33] Ibelo U, Green T, Thomas B et al. Ethnic differences in health literacy, self-efficacy, and self-management in patients treated with maintenance hemodialysis. Can J Kidney Health Dis 2022;9:1–12. 10.1177/20543581221086685 PMC8958521 35356537

[34] Havas K, Douglas C, Bonner A. Meeting patients where they are: improving outcomes in early chronic kidney disease with tailored self-management support (the CKD-SMS study). BMC Nephrol 2018;19:279. 10.1186/s12882-018-1075-2 PMC6195997 30342487

[35] Lai PC, Wu SFV, Alizargar J et al. Factors influencing self-efficacy and self-management among patients with pre-end-stage renal disease (pre-ESRD). Healthcare (Basel) 2021;9:266. 33801477 10.3390/healthcare9030266 PMC8000963

[36] Knowles SR, Ski CF, Langham R et al. Design and protocol for the Dialysis Optimal Health Program (DOHP) randomised controlled trial. Trials 2016;17:447. 10.1186/s13063-016-1558-z 27612446 PMC5018180

[37] Cukor D, Ver Halen N, Asher DR et al. Psychosocial intervention improves depression, quality of life, and fluid adherence in hemodialysis. J Am Soc Nephrol 2014;25:196–206. 10.1681/ASN.2012111134 24115478 PMC3871769

[38] Hedayati SS, Daniel DM, Cohen S et al. Rationale and design of A trial of Sertraline vs. Cognitive Behavioral Therapy for End-stage Renal Disease Patients with Depression (ASCEND). Contemp Clin Trials 2016;47:1–11. 10.1016/j.cct.2015.11.020 26621218 PMC4818161

[39] Berkhout-Byrne N, Gaasbeek A, Mallat MJK et al. Regret about the decision to start dialysis: a cross-sectional Dutch national survey. Neth J Med 2017;75:225–34. 28741581

[40] Parker WM, Ferreira K, Vernon L et al. The delicate balance of keeping it all together: using social capital to manage multiple medications for patients on dialysis. Res Social Adm Pharm 2017;13:738–45. 10.1016/j.sapharm.2016.07.008 27567742

[41] Clark S, Farrington K, Chilcot J. Nonadherence in dialysis patients: prevalence, measurement, outcome, and psychological determinants. Semin Dial 2014;27:42–9. 10.1111/sdi.12159 24164416

[42] Reid C, Seymour J, Jones C. A thematic synthesis of the experiences of adults living with hemodialysis. Clin J Am Soc Nephrol 2016;11:1206–18. 10.2215/CJN.10561015 27246010 PMC4934845

[43] Borsboom D. Measuring the Mind: Conceptual Issues in Contemporary Psychometrics. Cambridge: Cambridge University Press, 2005. 10.1017/CBO9780511490026

[44] Ladin K, Lin N, Hahn E et al. Engagement in decision-making and patient satisfaction: a qualitative study of older patients’ perceptions of dialysis initiation and modality decisions. Nephrol Dial Transplant 2017;32:1394–401. 10.1093/ndt/gfw307 27576590 PMC5837335

[45] Donald M, Kahlon BK, Beanlands H et al. Self-management interventions for adults with chronic kidney disease: a scoping review. BMJ Open 2018;8:e019814. 10.1136/bmjopen-2017-019814 PMC5875600 29567848

[46] Polenick CA, Perbix EA, Salwi SM et al. Loneliness during the COVID-19 pandemic among older adults with chronic conditions. J Appl Gerontol 2021;40:804–13. 10.1177/0733464821996527 33641513 PMC8238795

[47] Magadi W, Lightfoot CJ, Memory KE et al. Patient activation and its association with symptom burden and quality of life across the spectrum of chronic kidney disease stages in England. BMC Nephrol 2022;23:45. 10.1186/s12882-022-02679-w 35081904 PMC8793272

[48] Lightfoot CJ, Wilkinson TJ, Memory KE et al. Reliability and validity of the patient activation measure in kidney disease: results of rasch analysis. Clin J Am Soc Nephrol 2021;16:880–8. 10.2215/CJN.19611220 34117081 PMC8216620

[49] Van De Velde D, De Zutter F, Satink T et al. Delineating the concept of self-management in chronic conditions: a concept analysis. BMJ Open 2019;9:e027775. 10.1136/bmjopen-2018-027775 PMC6661649 31315862

[50] Haslam SA, McMahon C, Cruwys T et al. Social cure, what social cure? The propensity to underestimate the importance of social factors for health. Soc Sci Med 2018;198:14–21. 10.1016/j.socscimed.2017.12.020 29274614

