# Supplementary material for: A new self-management engagement scale for haemodialysis patients (PRIESM CKD-HD): development and validation
Source: Clin Kidney J. 2025 Dec 4;19(5):sfaf364. doi: 10.1093/ckj/sfaf364 (PMC13142153; doi:10.1093/ckj/sfaf364)
Supplement: sfaf364_Supplemental_File [file sfaf364_supplemental_file.pdf]

Supplemental table 1: Key terms

Term	Definition	Relationship with self-management
**Self-management**	Sub-set of self-care focussed on managing the impact of disease in terms of symptoms, treatment, physical, psychological, social consequences and lifestyle changes (Barlow et al., 2002; Wilkinson & Whitehead, 2009)	
**Self-efficacy**	"Beliefs in one's capabilities to organize and execute the courses of action required to produce given attainments." Bandura (1977) p. 3.<br><br>Disease-related self-efficacy is defined as an individual's confidence in their ability to adhere to treatment and manage disease-specific tasks (Lin et al., 2012).	A mechanism through which SM can be achieved (Peters et al., 2019). Perceived self-efficacy correlates with self-management behaviour (Curtin et al., 2008).
**Self-care**	Specific behaviours that individuals initiate and perform on their own behalf, with the intention of achieving, maintaining, or promoting optimal health and well-being. More recent definitions suggest informal caregivers may have a more central role (Denyes et al., 2001; Richard & Shea, 2011; Vellone et al., 2013).	Self-management as a sub-category of the broader concept of self-care (Matarese et al., 2018) but self-care does not incorporate the wider psychosocial impact of disease (Richard & Shea, 2011).
**Shared care**	A system of relational processes that includes providers-family-patient. May be emotional, practical, or informational support that enables individuals to be involved in their own treatment to the extent they wish (Fotheringham et al., 2017; 2021, Sebern & Woda, 2012).	Shared care better supports and empowers individuals to self-manage more broadly, beyond engagement with treatment (Wilkie & Barnes, 2019).
**Adherence**	The ability to follow a healthcare professional's recommendations on treatment regimens, regarding timing, doses, frequency, and periods of drugs-consumed but also diet and lifestyle (Deaton, 2000)	Adherence is a sub-category of self-management usually with a focus on clinical outcomes and treatment costs (Howren & Gonzalez, 2016).
**Patient activation**	Level of motivation, knowledge, skills, and confidence and individual has to make effective decisions to manage their health (Wagner, 1998 cited in Lightfoot et al., 2022; Hibbard et al., 2004; 2007).	Activation represents a narrow aspect of self-management (Lawless et al., 2021) and may be a precedent to self-management. Little is known about how to increase activation.

Supplemental table 2: Key themes form the qualitative analysis with illustrative quotes

Element	Themes	Description	Illustrative quotes
**Experience**	Dialysis process	The demands of dialysis such as the restrictions around 3-4 day a week schedules, the time lost, complications and the barriers to taking holidays.	"The only difficulty is you can't plan to do anything on the day you've got dialysis, it's a day swallowed up". (Male, White British, 86 years).<br><br>"Oh gosh, the time is waiting time, waiting to be picked up, now waiting to be collected and taken home, not a particularly good idea I suppose, but that is probably the most inefficient part of the whole of the setup, the transport" (Male, White British, 86 years)<br><br>"....it's just routine, I hate routine and this is the routine of three times a week. Like this morning I was just coming in and I thought, do you know what? I could just drive to the coast and the hell with it! But you can't do that..." (Male, White British, 74 years)<br><br>"We used to go across to the channel Islands quite a bit....just short breaks....But now even that's going to be a mission...unless I change my Saturday to Friday, but then that's if they've got the space here. Or organise it when we go away. But it's a lot of palaver just for a weekend away...." (Male, White British, 63 years)
	Symptoms	The experience of both physical and/or emotional symptoms, the burden and severity and how these impacts on self-managing behaviours. Includes the strategies that are used to cope with or prevent symptoms.	"I need something – the pain to go away, so I can do more, but this doesn't happen." (Female. Asian/Asian British, 55 years).<br><br>"Fatigue, just mind-numbingly, body-aching exhaustion" (Female, White British, 35 years).<br><br>"When I feel the nausea just like I said I do the tea early in the morning so that's fine. When I feel the tiredness the next morning unfortunately I don't have a strategy for it. I just know it will go away after a few hours and it does, so it's become part of my, you know, my life..." (Male, Black/Black British, 45 years).<br><br>"think to begin with I didn't handle things very well. I did get really depressed. I didn't want to leave my room, I didn't get washed or dressed, I didn't eat, I didn't do anything. I'd just be in bed all the time, and I think now it's like you try and handle things a bit different, so you don't get yourself into that point" (Female, Mixed race, 24 years).
	Other health conditions	The extent to which multimorbidity complicates managing of kidney disease,	"... The problem being, with the stoma – I have to drink fluids for it to work. And unfortunately, that takes me over, and, if I don't drink enough fluids, it erm constipates me. And to get it back

		particularly when multiple care providers are involved, and treatment recommendations/appointments may conflict. How does it impact on day to day priority setting and the capacity to engage with tasks of daily living?	working again, is heavy doses. So, I'm between the Devil and the deep blue sea." (Male, White British, 76 years).<br><br>"I was on medication for diabetes, blood pressure, glaucoma, this, that and the other. But they never specifically gave me anything for the kidney disease, whether it could have saved it or not I don't know, you know?" (Male, White British, 63 years).<br><br>"...there was a crash with me inside and it was my aortic valve, which is, well it's not doing its job... when they really got to grips that it is the aortic valve that needs replacing.... they concentrated far more on relevant things. I have my days off [dialysis] on Mondays and Wednesdays and of course then they have those days to call me in for tests, so I've been on the go either here or at hospital" (Male, White British, 86 years).
	Kidney transplant	Perceptions of the barriers to being on the transplant list, the experience of waiting, understanding how decisions are made and expectations.	"I was on the transplant list.....for 18 months, but, again, it's just I had cancer, thyroid cancer last year.... That affects me, but not the kidney, as such, and they've obviously taken me off the transplant list now. [...] what happens if you've had cancer, you go off it **[referring to list]** three to five years and that's it. I'm on the list, but I'm not active and I think that term is they used" (Female, White British, 62 years).<br><br>"I've been trying to get on the transplant list to get another kidney, but they obviously are not keen....they keep putting barriers in the way, and delaying tactics. I assume it's my age...I don't think they're very happy about doing it...So I'm stuck with the dialysis basically.... I mean I had an emergency hip operation and I was fine...I mean I went through the anaesthetic and all this...they're playing the long game..." (Male, White British, 74 years)<br><br>**[On the prospect of getting a transplant]** "...this is actually my last, last chance, and if I don't do it this time.... then I'm doing this (HD) forever..." (Female, ethnicity not coded, 51 years).
**Behaviours**	Management of daily tasks	Managing psychological responses to illness as well as practical aspects of managing illness either independently or with support from others, within the context of relationships, goals, values and life as usual.	"I got to wake up 40 minutes before just to start....getting dressed and that, you know, my wife has to help me with my shoes, with my socks because I can't bend over and do it. I pushed myself as much as I can, I push myself..." (Male, Asian/Asian British 48 years).<br><br>**[Talking about daughter-in-law]** "...so she does do most of the things anyway, I don't need to do anything... If I stand for about ten/15 minutes my whole back hurts, so in working-wise it's gone down, because I can't do everything by myself. It's like even when I have a shower, if you bend down and my back really hurts". (Female, Asian/Asian British, 55 years).

		“Cooking - I don't do much now. I'm a good cook, a very good cook, but not much....[who does the cooking?] No one. If my husband is at home, he does it, but mostly like takeaway or my sister has made something, and she asks us…” (Female, Asian/Asian British, 39 years)
Knowledge	If, when, and how patients seek information and how they use it. In what context is information sought and for what purpose, may be reassurance or in decision-making about treatment options etc.	“Well due to dialysis, sometimes it’ll crash the body, you know, when you take too much blood or fluids, again I had to do that again myself, - work it out, they didn’t tell me at the time – the nurses” (Male, Asian/Asian British, 48 years)<br><br>“I think you just have to kind of get on with it, and I've got a future to lead and I'm doing it for my family. So I think the information is there, but I think you personally have to go and find it” (Male, Asian/Asian British, 40 years).<br><br>**[self-management is]** "...being an expert in your own condition, and knowing as much as you can, and knowing an awful lot more than the medical professionals who only see you once a week. You're living it day to day, and so you have to self-manage to a certain extent" (Female, White British, 35 years).<br><br>“I do try and learn… as much as I can of self-care, that I'm doing. I've had about five attempts at lining the machine, and in terms of my medication, I take that religiously” (Male, White British, 72 years).
Coping strategies	Emotion and task focussed engagement strategies that are used to manage the illness within the context of everyday life.	“I’m used to now using the smallest cup in the house...In the beginning it’s a challenge, it’s difficult, but after a while I think your body adjusts and you just do it...” (Male, Black/Black British, 45 years).<br><br>“My big, big recommendation would be to take every form of help that is offered, because you can’t ever have too much help” (Female, White British, 35 years).<br><br>“I used to pace myself between the day and especially on the Wednesday and Fridays when I’ve had dialysis on Tuesdays and Thursdays. I don’t have to commute, so I just wake up, come downstairs, if I have the energy start work at 9, I don’t have it 10, then I’ll make sure I do my seven and a half hours” (Male, Black/Black British, 45 years).<br><br>**[Referring to dialysis unit] “**I’ve got this restriction of being here for about 10% of my time but I’m using that effectively, I think, so it’s doing stuff....I use the time....This is a different world and I don’t let it into my home life.” (Male, White British, 72 years).

	Meaningful life	Behaviours that help achieve a sense of balance. Maintaining activities that impact on wellbeing and provide a sense of purpose. Examples of how illness has been integrated into daily living a way that enables a person to maintain purpose and meaning.	"...because I'm restricted mobility, I took up electronics again.... I've got so many projects things I want to do, but I'm ending up making model aeroplanes for my grandchildren, and stuff like that.." (Male, White British, 74 years).<br><br>"I played golf up till Christmas, and the combination then of arthritis more [laughs] than anything to do with the kidneys. So my friends are still waiting for me to come back, but I haven't wanted to. I said to my consultant at Broomfield 'So I'll be able to tee off again?' he said, 'Yes, with a buggy perhaps'" (Male, White British, 86 years).
**Perceptions**	Beliefs about risks and consequences	Perceptions about the health risks linked to certain behaviours and adherence. Understanding the impact of behaviour in the long and short term.	"....in the beginning I had stopped and for 2 or 3 months I didn't have... But now I just have, like I would say one quarter of banana and like maybe 3 or 4 times a week, not more than that" (Female, Asian/Asian British 72 years).<br><br>"I do spoil myself now and again when my potassium's going a little bit low, but I wait until they tell me it's low and then I'll have a few peanuts, but other than that I have to leave them alone, you know? (Male, White British, 63 years).<br><br>"No. I had a little wine last night - champagne. I tend to think about a litre of water a day, or other liquids like tea. I don't drink coffee, and I don't drink beer. Occasionally, I might have some wine .... it affects me a bit and I don't feel good afterwards" (Male, White British, 72 years).<br><br>**[Talking about chips and mushrooms]** "I don't think I eat that amount to, you know, worry about, because erm maybe I'll be naughty like two days a week, five days a week I toe the line" (Male, White British, 76 years).
	Illness perceptions	Making sense of illness, its origins, and consequences. Impact of illness perceptions on physical function, adaption & acceptance	**[Talking about employer]** "Even if you're genuinely sick and its nothing to do with your kidney problem, they might go 'oh it's just kidneys again, oh yeah, he's sick again, oh yeah, sick again'. Last year when I was in hospital, I spent two weeks in hospital, I took that as holiday" (Male, Black/Black British, 45 years).

	and the impact of social situations.	“No I can’t have a transplant – not that I’m interested – I was on the list but because of the myeloma...I’d rather just finish off my life here, if I have to. I understand that....I could die any time. I’ve travelled the world in my work, so I don’t need to anymore” (Male, White British, 72 years).<br><br>“...it’s a way of life, you have to adapt, so my life now, my priority now is haemo and then life..” (Male, Black/Black British, 45 years).<br><br>“...and you know you’re stuck with it, and it’s that or you’re in a wooden overcoat, so.....But it does get me down occasionally.” (Male, White British, 74 years).
Self-identity	The intersection with illness and self and the degree to which illness is integrated into identity. Relational roles and social identity form core aspects of identity.	“I would say to my parents ‘I’m not doing this again, I’m not going to dialysis, I’m not having the operation. I give up, I’ve had enough. I don’t want to do it again, and I’m sick of being sick’ and I was really really bad...” (Female, Mixed race, 24 years).<br><br>“...they came around the other day **[referring to grandchildren]**...and the 22 year old went home and said to his Mum, he said...’do you know?....there’s nothing wrong with his brain (referring to patient)...he’s chatting away and he’s got all these ideas...’ and I said to (my wife)..well this is all failing but, thankfully, this is still going...” (patient points to head) (Male, White British, 74 years).<br><br>“....when I first went to the dialysis centre... I was waiting to be sent in, and I picked up the wrong leaflet and frightened myself to death! I went, oh, my God! And then put it down.....because it didn't really occur to me at that point that people do decide to stop. So, yeah, I quickly put that to the back of my mind, basically, but I'm luckier when you see some of the other people in the dialysis unit, and how poorly they are” (Female, White British, 62 years).<br><br>“Going out for like shopping with my husband or meeting friends...so now like social life is not there. [...] they live very far so it’s mostly the phone, that’s why sometimes I miss because I like talking to people and meeting them and the age doesn’t matter, whether it’s 3 year old or 80 year old” (Female, Asian/Asian British, 72 years).
Empowerment & control	Approaches to change, coping and enablers of self-management. A sense of control over the illness	"I don't have control over the illness, but I do have some control over the management" (Female, White British, 35 years).

		that is sufficient to allow involvement in the management of it if wanted. An ability to let go or accept things that can't be controlled.	"...and they do me a printout every month when they do bloods, and so I watch the potassium levels. If we... we eat out quite a lot, so I choose what I want and avoid the things that are wrong" (Male, White British, 86 years).<br><br>"You've just got to change your life, but at 76, you know, what do you change your life to? [laughs]" (Male, White British, 76 years).
	The future	Maintaining hope and anticipation for good things still to come. Anticipation and preparedness for the future, and reflections on a life lived.	"...somewhere in mind is even hope that one day I may come off". (Female, Asian/Asian British, 72 years).<br><br>"...you get very tired and you can't do the things you did before...I'm getting old...I mean, I'm 75. I can't complain, and I've had a good crack at it." [later goes on to say] [...] just the hope that at some stage you just might get a kidney transplant, and you can start living hopefully a normal life. And they keep saying to me, oh well, because it won't increase your life, and it's no, but it will improve the quality" (Male, White British, 74 years).<br><br>"...Pre-dialysis I think it's a really bad time in your life because you are not dialysing and there's a hope that maybe your kidney situation will turn around" (Male, Black/Black British, 45 years).
**Others**	Impact on family	The degree to which family members provide both task-based and emotional support. The impact of this on those caring and the person supported. Examples, enabling self-management, increased family conflict, feelings of being a burden, or where support is perceived as negative and may affect the patient's ability to self-manage.	[In reference to husband] "....it is because of him that everything goes on in the house, the shopping, if my son and daughter has come he will look after them as well and make them tea or cook a bit of rice and all those things, he does a lot. I think without him I wouldn't be anywhere" (Female, Asian/Asian British, 72 years).<br><br>"It's put a huge strain on the relationship....and we've both had different experiences of the same event. And we've processed it at different times, and we've processed it differently......I think he's looking forward to a time when we will be more of an equal partnership"(Female, White British, 35 years).<br><br>"My wife had no choice but to go to work" (Male, Asian/Asian British, 40 years).<br><br>[In reference to wife] "...has become much more of a carer, so she's had to take on stuff that I would normally, like, I don't know, put the bins out or whatever....which is a shame really because I think she gets weary..." (Male, White British, 74 years).
	Healthcare providers	Support offered by clinical staff to promote and support self-	In relation to a procedure – "he came and sat down with me, and we both talked to each other, and then he said 'I don't think I can actually do this. I don't think it's going to improve you

	management that goes beyond information sharing and medical support of dialysis to wider goal setting, problem-solving support, joint decision making and more holistic support. Patient's perception of the quality, availability, and experience of support.	condition in any way'….but he was there and he was ready to give me the treatment" (Male, Asian/Asian British, 40 years).<br><br>[Decision to go on dialysis] "I remember this one time I was travelling and the nurse said to me…this is going to blow up…you need to start….when I look back I think it was very poor judgement on my part and it was because I was being defensive about it….I just didn't want to start…" (Male, Black/Black British, 45 years).<br><br>[in response to being asked if there was more to say] "no, unless you've got any influence about me starting home dialysis [you'd be interested?] Yeah, more than interested in that, at my age. I mean I'm quite a fit person for my age, I'm quite alert. [….] Just kind of waiting. Somebody did speak to me right at the beginning, but I haven't heard anything. [Asked about the benefits] I can keep an eye on my wife". (Male, White British, 76 years).<br><br>"And it's the worrying about it, and if you worry about something the best thing to do is ask the doctor, and they'll advise you." (Female, Asian/Asian British, 55 years).

Supplemental table 3: Word changes post I-CVI round 2, based on decisions by research team and comments made by consensus panel

Original wording	Revised wording	Comments from consensus panel
I have come to accept the long-term nature of my kidney problem.	I have come to accept the life-long nature of my kidney problem	I know long-term is always used but it's actually life-long?
Despite the dialysis I try to live as normal a life as possible	Despite being on dialysis I try to live as normal a life as possible.	The phrase 'the dialysis' seems strange and I wonder if this needs to be re-worded. E.g. 'being on dialysis'?
I feel I have enough control over my dialysis treatment	I feel I have control over my dialysis treatment.<br><br>Anchors altered to reflect degree 'no control'/'enough control'	Overlap with the previous question 'Since starting dialysis, I feel less able to control the important things in my life'.
I feel I have some control over my kidney disease symptoms	I feel I have control over my kidney disease symptoms.	As for above 'no control'/'some control'
I play a large role in managing my illness	I have an important role in managing my illness.	not sure how clear the term 'large role' is. Suggest: 'I take responsibility for managing some aspects of my illness'
I [often] feel [that] I cannot cope with all the things I need to do.	I feel that I cannot cope with all the things I need to do.	The brackets are confusing, I would not use often, it doesn't need to be often for it to be important or worthy of discussion -maybe sometimes is better?
[At times], I feel overwhelmed by my illness(es)	I feel overwhelmed by my kidney disease.	I think it should specifically be asking about kidney disease- it's very hard to answer a question about many diseases, as a person might be overwhelmed by some and not others.
Being on dialysis makes it difficult for me to access treatment or attend appointments for my other health problems	Being on dialysis makes it difficult for me to get the care I need for my other health problems.	Could we take out 'access treatment or' and it mean the same thing? Add sometimes?
I often get conflicting advice from the different specialists I need to see.	I get conflicting advice from the different specialists I see.	shouldn't it be more about all the 'healthcare professionals' as are GPs defined as specialists? What about diabetes nurse specialists etc.
I sometimes avoid social situations [where there will be food and drink] as I don't want others to know about my kidney problems	I avoid social situations [where there will be food and drink] as I don't want others to know about my kidney problems.	Use of "food and drink" too directive. May be other reasons, exclude.
My kidney disease has changed how I see myself	I see myself more negatively since the kidney disease.	Research team felt this should be more specific.

I feel I have to keep on going for my family	The main reason I keep going is for my family and/or people close to me.	family and people close to me? Some people may not have close family but may have close friends
There have been times when I have shortened my dialysis time due to family or work commitments	I have shortened my dialysis time due to family or work commitments.	Research team felt this could be more concise without losing meaning
I feel like a burden on my family	Sometimes I feel like a burden on my family.	Might be upsetting for the patient to answer.
I feel that I have a good understanding of how my illness will progress	I understand how my illness will progress.	Research team felt this could be more concise without losing meaning.
I look for information or talk with a pharmacist about my medications.	I understand what my medications are for.	Split sentence, why pharmacist in particular?
I've tried to learn more about the dialysis machine and other technical topics.	I've tried to learn more about my dialysis.	Suggested 'technical issues related to my dialysis treatment'. Research team decided to simplify.
My kidney disease interferes too much with my life	My kidney disease interferes with my life.<br><br>Anchors altered to reflect 'too much'/'not at all'	It does for all kidney patients.
There is someone in my life whose advice and encouragement helps me manage my kidney disease day to day.	There is someone in my life whose advice and/or encouragement helps me manage my kidney disease day to day.	Amendment made by research team, may not be both advice and encouragement.
One way or another I can manage most of the day-to-day tasks such as washing, dressing, jobs around the house.	One way or another I can manage most of the things I need to do day-to-day.	Research team felt this could be more concise without losing meaning.
I am still able to do the things I used to enjoy before I had to start on dialysis.	There are things that I miss that I used to enjoy before I had to start on dialysis.	Change made by research team, they felt changing the emphasis improves the question.
My kidney disease prevents me from taking part in social activities as much as I would like.	My kidney disease prevents me from taking part in social activities	Research team felt this could be more concise without losing meaning.
I take responsibility for parts of my care or treatment that might otherwise be performed by renal staff.	I take responsibility for parts of my care that might otherwise be done by renal staff.	Research team felt language could be simplified.
Symptoms often interfere with the way I would like to live my everyday life.	My symptoms interfere with the way I would like to live my everyday life.	I would begin with MY symptoms.

	Anchors altered to reflect 'Hardly ever'/'often'	
Sometimes my feelings of distress are overwhelming	Some days my feelings of distress are overwhelming.	Research team felt this question should be more specific.
Tiredness stops me from getting on with life the way I would like.	Tiredness stops me from getting on with life.	Research team felt this could be more concise without losing meaning.
I decide which problems to report to doctor / nurse and which to handle on my own	I know which problems or symptoms to consult the renal team about.	Maybe always say care team/renal team, or are you specifically interested in doctor and nurse? Suggested wording "I decide which problems I discuss with the renal team and which to handle myself"
When discussing treatment or management options I feel that my views are fully considered by the renal team.	When discussing treatment options, I feel that my views are considered by the renal team.	Be consistent for care team and renal team.<br><br>Research team felt this could be more concise without losing meaning.
I feel I wasn't as involved in the decision to start dialysis as I would have liked [because I was too distressed, or unwell]	I feel I wasn't as involved in the decision to start dialysis as I would have liked.	I wonder why you have added the section in brackets - it makes it more difficult to answer the question
When I need to make a treatment decision, I talk it over with someone I am close to.	When I need to make a treatment decision, I talk it over with someone I trust.	Medical, Family, Friend? Suggested wording "When I need to make a treatment decision, I can talk it over with someone I trust"
Haemodialysis treatment makes keeping working difficult	Haemodialysis treatment makes working difficult.	Question phrase isn't completely clear to read - Being on dialysis makes it difficult to work (paid employment) OR My haemodialysis treatment makes it difficult to keep working.
My ability to do paid work is really affected by my health.	My ability to work is really affected by my heath.	Do you want to specify kidney disease? "My ability to work is affected by my haemodialysis treatment"

Supplemental table 4: Cognitive pre-testing led to six items removed.

Dropped item	P1	P2	P3	P4	P5	P6	P7	P8	P9	P10	P11
There is someone in my life whose advice and/or encouragement helps me manage my kidney disease day to day.	4		1			7	7	7	7	7	1
I keep a careful watch on what I eat and drink so that I can make changes if necessary.	4		6			7	4	1	5	6	N/A
I seek the company of friends, family and/or other patients.	7		2			2	7	7	DK		-
The renal team support me with setting and achieving my goals.		N/A		-	-				-		-
When I need to make a treatment decision, I talk it over with someone I trust.	6		6	4		7	1	1	2	1	
I feel confident doing the dialysis myself.		7		1	N/A	1			1	1	

Note – not all participants tested each item, grey indicates where this is the case. The number represents the score (1-7) that the respondent allocated.

Supplemental table 5: Distribution of scale items

	Obs.	[a]Mean (S.D)	Median	% score 1	% score 7	[a]Don't know (%)	[a]N/A (%)	[a]Missing (%)
**Item 1**	358	5.99 (1.48)	7	1.45	57.6	6 (1.68)	8 (2.2)	5 (1.4) [c]
**Item 2**	357	6.22 (1.35)	7	2.54	61.3	2 (0.56)	2 (0.56)	6 (1.7)
**Item 3**	354	6.17 (1.30)	7	1.73	57.0	5 (1.41)	3 (0.85)	9 (2.5)
**Item 4**	356	6.02 (1.49)	7	2.31	55.3	6 (1.69)	3 (0.84)	7 (1.9) [c]
**Item 5**	353	5.25 (1.73)	6	5.1	33.1	3 (0.85)	2 (0.57)	10 (2.8)
**Item 6**	355	5.82 (1.60)	7	3.42	51.0	3 (0.85)	2 (0.56)	8 (2.2)
**Item 7**	356	5.65 (1.57)	6	2.82	42.3	0 (0)	1 (0.28)	7 (1.9) [c]
**Item 8**	357	5.03 (2.07)	6	11.2	35.7	3 (0.84)	7 (1.96)	6 (1.7) [c]
**Item 9**	351	3.13 (2.25)	2	40.4	13.7	8 (2.28)	60 (17.1)	12 (3.3)
**Item10[r]**	355	2.56 (1.69)	2	3.7	38.9	2 (0.56)	3 (0.85)	8 (2.2) [c]
**Item11**	355	4.47 (2.24)	5	17.3	29.6	1 (0.28)	13 (3.66)	8 (2.2) [c]
**Item 12**	352	5.71 (1.45)	6	1.78	41.3	16 (4.55)	0 (0)	11 (3.0) [c]
**Item 13**	355	5.09 (1.82)	6	7.1	28.0	1 (0.28)	0 (0)	8 (2.2)
**Item 14**	354	4.88 (1.78)	5	6.3	25.7	2 (0.56)	2 (0.56)	9 (2.5) [c]
**Item 15**	347	5.76 (1.41)	6	1.5	40.2	2 (0.58)	3 (0.86)	16 (4.4)
**Item 16**	349	5.40 (1.77)	6	5.3	40.2	7 (2.01)	21 (6.02)	14 (3.9)
**Item 17**	345	3.90 (2.48)	4	30.0	28.2	7 (2.03)	9 (2.61)	18 (5.0)
**Item 18**	341	4.01 (2.36)	4	29.9	20.7	17 (4.99)	61 (17.9)	22 (6.1)
**Item 19**	349	5.31 (1.74)	6	6.0	31.5	10 (2.87)	7 (2.01)	14 (3.9)
**Item 20**	350	6.58 (0.99)	7	1.2	76.6	5 (1.43)	3 (0.86)	13 (3.6)
**Item 21**	349	3.53 (1.96)	3	23.0	10.9	0 (0)	2 (0.57)	14 (3.9)
**Item 22**	349	4.89 (1.93)	5	8.5	29.5	1 (0.29)	5 (1.43)	14 (3.9)
**Item 23**	348	5.51 (1.45)	6	1.7	34.4	2 (0.57)	0 (0)	15 (4.1)
**Item 24**	351	5.28 (1.80)	6	5.5	36.4	9 (2.56)	16 (4.56)	12 (3.3)
**Item 25**	348	4.41 (2.13)	5	14.9	22.5	3 (0.86)	32 (9.2)	15 (4.1)
**Item 26**	349	4.76 (2.35)	5	20.2	37.6	5 (1.43)	17 (4.87)	14 (3.9)
**Item 27**	351	5.88 (1.75)	7	5.3	59.4	5 (1.42)	4 (1.14)	12 (3.3)
**Item 28**	352	4.70 (2.15)	5	15.4	31.0	5 (1.42)	2 (0.57)	11 (3.0)
**Item 29**	353	4.73 (2.07)	5	11.8	28.7	2 (0.57)	3 (0.85)	10 (2.8)
**Item 30[r]**	353	3.53 (2.08)	3	14.0	23.3	4(1.13)	6 (1.70)	10 (2.8)
**Item 31**	353	4.32 (2.21)	4	18.2	23.5	6 (1.70)	6 (1.70)	10 (2.8)
**Item 32**	351	5.28 (1.82)	6	6.2	37.6	6 (1.71)	7 (1.99)	12 (3.3)
**Item 33**	353	5.07 (2.16)	6	12.0	42.8	7 (1.98)	13 (3.68)	10 (2.8)
**Item 34**	353	6.13 (1.30)	7	1.1	57.3	1 (0.28)	2 (0.57)	10 (2.8)
**Item 35**	354	5.85 (1.51)	6	3.5	45.7	5 (1.41)	1 (0.28)	9 (2.5)
**Item 36**	352	5.23 (1.90)	6	6.9	35.1	7 (1.99)	12 (3.41)	11 (3.0)
**Item 37**	353	5.78 (1.56)	6	2.9	44.5	4(1.13)	5 (1.42)	10 (2.8)
**Item 38[r]**	352	4.01 (2.10)	4	20.3	17.1	1 (0.28)	6 (1.70)	11 (3.0)
**Item 39**	339	4.79 (1.98)	5	9.2	28.6	6 (1.77)	48 (14.16)	22 (6.1) [c]
**Item 40**	347	4.26 (2.05)	4	13.4	20.1	2 (0.58)	3 (0.86)	16 (4.4) [c]
**Item 41**	347	5.26 (1.86)	6	7.0	36.0	2 (0.58)	3 (0.86)	16 (4.4) [c]
**Item 42**	350	6.01 (1.55)	7	4.1	57.2	3 (0.86)	8 (2.29)	13 (3.6) [c]
**Item 43**	348	6.11 (1.35)	7	1.5	57.8	1 (0.29)	2 (0.57)	15 (4.1) [c]
**Item 44**	352	5.52 (1.80)	6	4.5	43.9	9 (2.56)	8 (2.27)	11 (3.0) [c]
**Item 45**	348	4.14 (2.26)	4	21.4	23.6	11 (3.16)	16 (4.60)	15 (4.1) [c]
**Item 46**	350	5.82 (1.92)	7	7.9	61.8	0 (0)	10 (2.86)	13 (3.6) [c]
**Item 47**	348	4.68 (2.16)	5	12.7	32.0	4 (1.15)	6 (1.72)	15 (4.1) [c]
**Item 48**	348	4.78 (2.23)	6	14.7	34.7	3 (0.86)	20 (5.75)	15 (4.1) [c]
**Item 49**	348	6.17 (1.30)	7	1.2	58.8	7 (2.01)	0 (0)	15 (4.1) [c]
**Item 50**	350	5.87 (1.78)	7	5.6	59.1	3 (0.86)	11 (3.14)	13 (3.6)
**Item 51**	351	4.78 (1.94)	5	8.4	25.9	3 (0.85)	4 (1.14)	12 (3.3)

**Item 52**	351	5.18 (1.98)	6	9.7	37.3	7 (1.99)	14 (3.99)	12 (3.3)
**Item 53**[r]	352	2.88 (1.84)	2	6.6	30.0	2 (0.57)	3 (0.85)	11 (3.0)
**Item 54**	350	5.66 (1.55)	6	2.2	39.9	16 (4.57)	19 (5.43)	13 (3.6)
**Item 55**	349	4.61 (2.08)	5	11.5	27.1	6 (1.72)	4 (1.15)	14 (3.9)
**Item 56**	348	3.94 (2.38)	4	30.6	22.2	4 (1.15)	61 (17.53)	15 (4.1)
**Item 57**	347	5.96 (1.52)	7	3.5	54.0	0 (0)	6 (1.73)	16 (4.4)
**Item 58**	347	5.77 (1.47)	6	2.4	43.8	6 (1.73)	3 (0.86)	16 (4.4)
**Item 59**	345	3.82 (2.00)	4	16.2	12.6	0 (0)	238[b] (69.0)	18 (5.0)
**Item 60**	343	3.30 (2.19)	3	33.3	13.7	1 (0.29)	230[b] (67.1)	20 (5.5)

[a] Data are presented as *n* (%), include response 1-7 only, don't know, N/A and missing excluded.

[b] These items may be perceived as relevant only to respondents currently in employment (17.3% of the sample).

[c] Missing values for this item may not be missing at random when considering the sex of the respondent.

[r] Item reverse scored

Supplemental table 6: Principal component factor analysis, unrotated, number of factors unspecified.

FACTOR	EIGENVALUE	DIFFERENCE	PROPORTION OF VARIANCE	CUMULATIVE
**FACTOR 1**	12.95	6.90	0.216	0.216
**FACTOR 2**	6.05	1.55	0.101	0.317
**FACTOR 3**	4.50	1.21	0.075	0.392
**FACTOR 4**	3.29	0.19	0.055	0.447
**FACTOR 5**	3.10	0.60	0.052	0.498
**FACTOR 6**	2.50	0.21	0.042	0.540
**FACTOR 7**	2.29	0.18	0.038	0.578
**FACTOR 8**	2.11	0.20	0.035	0.613
**FACTOR 9**	1.91	0.01	0.032	0.645
**FACTOR 10**	1.90	0.19	0.032	0.677
**FACTOR 11**	1.71	0.18	0.029	0.706
**FACTOR 12**	1.53	0.18	0.026	0.731
**FACTOR 13**	1.35	0.05	0.023	0.754
**FACTOR 14**	1.30	0.15	0.022	0.775
**FACTOR 15**	1.15	0.02	0.019	0.795
**FACTOR 16**	1.14	0.11	0.019	0.814
**FACTOR 17**	1.03	0.11	0.017	0.831

Supplemental table 7: Rotated factor loadings (3 factors specified)

Model	Retained	Added	Removed	Items	χ2	CFI	TLI	RMSEA	SRMR	α
Model 1				36	1603.016*	0.794	0.781	0.074	0.083	0.93
Model 2		Item 5	Item 2, Item 42	35	1518.964*	0.790	0.776	0.074	0.082	0.93
Model 3	Item 5		Item 35, Item 48	34	1518.964*	0.790	0.776	0.074	0.082	0.93
Model 4	Item 5		Item 7, Item 52	32	1267.015*	0.804	0.790	0.075	0.079	0.93
Model 5	Item 5	Item 7	-	33	1436.321*	0.795	0.781	0.075	0.080	0.94
Model 6			Item 7, Item 43	29	1156.915*	0.816	0.801	0.073	0.078	0.92
Model 7			Item 5	30	1013.656*	0.837	0.824	0.070	0.072	0.92
Model 8		Item 5	Item 23	30	1080.006*	0.818	0.803	0.073	0.078	0.92
Model 9	Item 5		Item 19	29	983.725 *	0.828	0.813	0.072	0.076	0.92
Model 10		Item 19	Item 8	29	1042.317*	0.814	0.798	0.076	0.080	0.92
Model 11		Item 8	Item 19	29	983.725 *	0.828	0.813	0.072	0.076	0.92
Final			Item 5, Item 53, Item 55	26	703.033*	0.869	0.857	0.066	0.071	0.91

*<0.001

Sample size varied from 311 – 317 respondents across all iterations

Model 1: ***F1*** (25, 29, 51, 13, 16, 38, 14, 53, 28, 21, 55, 22, 40, 10, 7, 4) ***F2*** (2, 3, 4, 6, 1, 15,54, 44, 35) ***F3*** (19, 41, 43, 8, 47, 23, 42, 26, 58, 52, 33)

Final Model: ***F1*** (25, 29, 51, 13, 16, 38, 14, 40, 28, 21, 22, 47 10) ***F2*** (8, 3, 6, 4, 15, 44, 54, 1, 33) ***F3*** (42, 58, 26, 52)

Supplemental table 8: Summary of items that were removed between model 1 and final model.

Scale ref	Factor	Content (abbreviated items)	Issue
Item 7	F1	Facing the challenges of kidney disease.	Cross loading across all 3 factors
Item 48	F1	Interference with relationships.	Weak loading <.4
Item 53_R	F1	Tiredness stops 'getting on' with life.	Dropped in final model due to low correlation with 3 other items within FI (corr <.25), <.4 item test & rest correlation and moderate factor loading (.5)
Item 55	F1	Difficulties for those who are close to me.	Dropped in final model to reduce items in F1, omission improved fit statistics
Item 2	F2		Inter-item correlation with items 3, 4, 6 and 1 (>.6)
Item 5	F2	Control over symptoms.	Cross loading across F1 and F2
Item 35	F2	Knowing when to consult the renal team about problems.	Weak loading <.4
Item 19	F3	Control over dialysis treatment.	Moderately high inter-item correlation with Items 19 and 23 (>.5)
Item 23	F3	Knowing enough to deal with day-to-day problems.	Weak loading <.4 and moderate correlation with item 19
Item 41	F3	Having good things to look forward to.	Weak loading <.4
Item 43	F3	Despite dialysis, trying to live a normal life.	Cross loading across F1 and F3

Supplemental table 9: Mean plasma phosphate level by PRIESM CKD-HD domain

	Overall	Managing	Communication	Clinical
**Phosphate level**				
Below 1.4 mmol/L	5.12 (SD±1.04)	4.60 (SD±1.37)	5.97 (SD±0.94)	5.45 (SD±1.26)
1.4 to 2 mmol/L	5.10 (SD±1.03)	4.61 (SD±1.29)	5.92 (SD±1.10)	5.36 (SD±1.24)
Above 2 mmol/L	4.56 (SD±1.20)**	4.00 (SD±1.44)*	5.24 (SD±1.46)**	5.19 (SD±1.28)

Baseline category: within range 1.4-2 mmol/L
$*p \leq 0.05$ $**p \leq 0.01$

Supplemental figure 1: Topic guide outline for interview

Note on formatting - main questions in bold, sub-questions italicised, prompts as bullet points.

Demographic information (collected via clinical records as part of the eligibility process)

Age, sex, ethnicity, and time on dialysis (vintage)

Topic 1: Self and experience

1 I wonder if we might begin with you telling me a little bit about you and your kidney disease. Please tell me about yourself.

Prompts:

- Overview age/family/interests/important things in life
- CKD diagnosis – when,
- History on dialysis; years?

Topic 2: Symptoms and treatment

2 And to help me to understand what living with kidney disease is like, can you tell me about the symptoms you experience?

2.1 How do you deal with your symptoms?
2.2 *What do you do when symptoms are really bad?*

Prompts:

- Consequence of the illness,
- Dialysis specifically
- Including other comorbidities
- Who supports them?
- Who do they discuss with?
- What support do they have?

c. *Some people describe dealing with quite difficult emotions at different stages of their kidney disease, how do you cope with any you experience?*

3 Can you tell me about your experience of treatment?

3.1 If you wanted to change your treatment or stop dialysis, what would you do?

Prompts

- How does it impact their life?

Topic 3: Daily managing

4 **How do you look after yourself day to day?**

4.1 Who helps?
4.2 What things cause you the most worry? These things may or may not be related to your kidney problems.

4.3 And what things are you happy that you've got under control?

Prompts

- In relation to kidney disease but also beyond.

5 Can you tell me about the types of advice are you given about how to stay well?

5.1 *What are your thoughts on it?*
5.2 *Does this advice help you live the life you want for yourself?*

Prompts

- Who gives what advice?
- Are the messages coherent and in agreement?
- The impact or consequence of doing or not doing these things?

6 It sounds like there is a lot involved in managing kidney disease – is there anything you do or don't do that you know might help you at all? Are there any things you do that may not be the best thing for your kidney care?

Prompts

- What are the barriers?

7 What would you recommend someone else do to manage some of the experiences and symptoms you've talked about?

7.1 *What would you say is the one thing you do best to help you manage your kidney disease and treatment?*

Topic 4: Treatment decisions

8 Can you tell me about any treatment decisions you've had to make since having kidney disease?

8.1 *Are there any decisions you'd go back and change if you could?*

Prompts

- How were the decisions made?
- Who with?

Ending

9 Is there anything else you would like to say about what matters to you that hasn't been discussed?

9.1 Do you have any other comments about what we have discussed or feedback on the interview process?

Supplemental figure 2: PRIESM CKD-HD – 26-item final scale

	THEME: DAILY MANAGING AND IMPACT	RESPONSE OPTIONS	
**Q29**	I find it difficult to look after myself the way I would like to.	Always	Never
**Q38**	Some days I feel distressed.	Hardly ever	Often
**Q25**	I have other health issues as well as my kidney disease and I find it difficult to cope with them all.	Very difficult	Not at all difficult
**Q51**	I feel that I cannot cope with all the things I need to do.	Often	Never
**Q40**	I sometimes feel overwhelmed by my kidney disease.	Strongly Agree	Strongly Disagree
**Q14**	I find it hard to cope with my kidney disease.	Completely	Not at all
**Q28**	I am angry about my kidney disease.	Very Angry	Not Angry at all
**Q47**	I see myself more negatively since I've had kidney disease.	Strongly Agree	Strongly Disagree
**Q21**	My kidney disease interferes with my life.	Too much	Not at all
**Q22**	I have activities and interests that keep me busy.	Strongly Disagree	Strongly Agree
**Q13**	Day to day I can manage most of the things I need to do.	Strongly Disagree	Strongly Agree
**Q10**	My symptoms interfere with the way I would like to live my everyday life.	Never	Always
**Q16**	Being on dialysis makes it difficult for me to get the care I need for my other health problems.	Often	Never
**Q8**	I feel more isolated than I did before I started dialysis.	Strongly Agree	Strongly Disagree
	**THEME: COMMUNICATION**		
**Q3**	When I talk to the renal team about my care I'm treated as a whole person rather than someone with an illness.	Never	Every Time
**Q6**	The renal team review my health and discuss any changes with me.	Hardly Ever	Enough
**Q4**	I have someone who will listen to me when I need to talk.	Not at all true	Completely True
**Q15**	I get the support I need to manage my health and treatment.	Strongly Disagree	Strongly Agree
**Q1**	I'm able to ask a doctor for more information if I don't understand what he or she said.	Never	Every Time
**Q54**	When discussing treatment options, my views are considered by the renal team.	Hardly Ever	Often
**Q44**	I am confident that there is good communication between all the specialists I see.	Not Confident	Very Confident
**Q33**	I wasn't as involved in the decision to start dialysis as I would have liked.	Completely True	Not at all true
	**THEME: CLINICAL CARE**		
**Q26**	I check settings on the dialysis machine to make sure they are the same as usual.	Never	Always
**Q58**	I have an important role in managing my illness.	Strongly Disagree	Strongly Agree
**Q42**	I help decide how much fluid should be taken off.	Never	Always
**Q52**	The renal team encourage me to be involved in my own care.	Never	Always

Note: scale items are presented by magnitude of factor loading with themes/domains as determined by exploratory factor analysis (orthogonal varimax rotation)
